# Assessing the Effectiveness of Social Indices to Measure the Prevalence of Social Isolation in Neighbourhoods: A Qualitative Sense Check of an Index in a Northern English City

**DOI:** 10.1007/s11205-017-1812-0

**Published:** 2017-12-02

**Authors:** Andrea Wigfield, Sarah Alden

**Affiliations:** Department of Sociological Studies, University of Sheffield, Northumberland Road, Sheffield, S10 2TU UK

**Keywords:** Social isolation, Index, Indicators, Ageing, Neighbourhood, Wellbeing

## Abstract

Tackling the many negative health effects of social isolation has been identified as a policy priority in the UK and consequently many local authorities are developing strategies to ascertain its prevalence through the development of social indices. This paper provides a novel assessment of the emerging approach of developing indices to identify social isolation. It provides an overview of a selection of indices being developed by local authorities across England; considers the validity of such quantitative indices; and explores the extent to which more in-depth qualitative data collected at a neighbourhood level is additionally required. It draws on evidence of a social isolation index for older people created by a northern English local authority, assessing its validity through a qualitative sense check; an innovative approach which has not been attempted elsewhere. The paper contributes important knowledge to the growing literature in this field by further developing understanding around the most effective ways of identifying, measuring, and understanding social isolation at a local level. Our findings indicate that an index, alone, is insufficient to fully capture the multifaceted nature of social isolation as relevant indicators, unique to local spaces, which cannot easily be measured quantitatively, are often excluded. The paper offers a significant and original contribution to the debate for both academics who wish to gain a greater understanding of the role indices can play in identifying those most at risk of social isolation, as well as for policy makers and practitioners who are currently grappling with this challenging concept.

## Introduction

As the population ages and growing numbers of people are living alone, social isolation in later life is emerging as a major issue (Bardsley et al. 2011; Jowit 2013; Victor 2010). Based on data from the Office of National Statistics, Hawton et al. (2011) estimate that approximately 1.3 million older people in the UK are possibly ‘severely socially isolated’. Social Isolation can have adverse effects on health and wellbeing (Findlay 2003; Office of the Deputy Prime Minister 2006). For example social isolation has been linked to increased mortality (Holt-Lunstad et al. 2010; Steptoe et al. 2013) and morbidity (Bosworth and Schaie 1997), with those who experience social isolation being between two and five times more likely to die prematurely, than those who have strong social ties (Marmot et al. 2010: 138). Social isolation also elevates the likelihood of developing limiting health conditions (Holt-Lunstad et al. 2010). Conversely, social participation helps to maintain physical and mental wellbeing in later life (Bolton 2012; Reynolds et al. 2015) creating a ‘buffer’, which can help limit the impact of stressful life events (Farmer and Sundberg 2010), such as bereavement (Wenger 1997: 311). Research has also shown that increasing participation may lessen GP visits, reduce the need for treatment to deal with depression (Windle et al. 2011), and may act as a protective factor against cognitive impairment (Marmot et al. 2010). Therefore, reducing social isolation can lead to improved health and well-being, triggering potential cost savings for health and social care services, and consequently has been identified as a policy priority in the UK (DH 2014).

The negative impact of social isolation on health and quality of life, coupled with the current policy direction around the promotion of wellbeing, and the Big Lottery’s £78 m Fulfilling Lives: Ageing Better programme aimed at reducing social isolation among older people in 14 areas of England, has led some local authorities to identify and target social isolation ‘hotspots’ in their respective localities through the development of social isolation indices. Given that these ‘hotspots’ are potentially being used to direct resources to tackle social isolation, it is important to assess the extent to which the indices are a robust approach. Using evidence gained through a study involving a qualitative ‘sense check’ of an index created by a northern local authority (Leeds City Council), this paper considers the extent to which social indices can offer an effective approach to identifying and measuring the prevalence of social isolation. It assesses the degree to which such indices are sufficient alone, and the extent to which they may benefit from supplementary qualitative data.

It is important to point out at this juncture that a small number of Third Sector organisations have also developed social isolation indices, and although a discussion of this is beyond the scope of this paper, the findings discussed herein may also be usefully applied to indices developed by these organisations. The findings will also be of interest to public and third sector organisations, both in the UK and further afield, who are either looking to, or in the process of, developing indices to measure related concepts such as loneliness and wellbeing. Whilst the research outlined here was based on an index to identify and measure social isolation among older people only, the principles discussed can be applied to indices for any age group (though, of course, resultant indices may include different indicators, dependent on the group being assessed).

This paper firstly situates the term social isolation within broader debates, providing an overview of the main indicative components identified in the existing literature. It then turns to the social isolation indices being developed by local authorities, before discussing the index created by Leeds City Council. The paper concludes with a discussion exploring the usefulness of an index of social isolation and the additionality offered by supplementing a social isolation index with qualitative information gathered at a local level.

## Social Isolation: How is it Defined and Measured?

### What is Social Isolation?

For the purposes of this paper social isolation is defined as *‘an objective measurable state of having minimal contact with other people, such as family, friends or the wider community’* (Cattan 2002: 11). Social isolation is viewed as a multidimensional construct; where it can be transitory (i.e. at onset of retirement), or more persistent and longstanding. Furthermore, a person may be assessed as isolated in some aspects of their life, but not in others. For example they may have regular contact with family (social networks), yet be disconnected from their community or peers (Jivraj et al. 2012).

It is important to position social isolation within the broader conceptual literature which attempts to understand the impact of social interaction within the various layers of contemporary society (i.e. at the familial and community level and/or within wider economic, political, civic and cultural contexts). Social isolation in many cases is either central to, or at least forms part of, frameworks which encompass social interaction (Zavaleta et al. 2014). Examples of such frameworks include social connectiveness, social capital and social cohesion alongside more negatively phased concepts such as alienation, social exclusion, and particularly loneliness, the latter of which is frequently conflated with social isolation.

Loneliness and social isolation share similar risk factors (Local Government Association 2016) with a lack of social connectiveness forming the overarching focus of the two terms. It has been asserted that being socially isolated may suggest a heightened likelihood of experiencing the loneliness (Local Government Association 2016) and that reducing social isolation is the most effective way of addressing loneliness (Age UK 2010). Therefore, linking the concepts together, at least at first, appears to makes ‘intuitive’ sense (Bristol City Council 2014). In fact, in defining social isolation some commentators have attempted to mesh the two terms, such as referring to the quantity ‘and’ quality of social contacts (Zavaleta et al. 2014), or Fine and Spencer’s (2009) ‘social’ and ‘affective’ social isolation.

However, we suggest that the two concepts are different and should be handled separately. This is particularly important as an individual who is not social isolated may nevertheless feel lonely and, of course, vice versa (Davidson and Rossall 2014). Related to this, potential solutions differ, as the support required by those who are socially isolated varies from that needed by those who feel lonely (Bolton 2012). Social isolation is also more amenable to measurement than loneliness, due to its widely accepted conceptualisation as an objective assessment of social contacts (i.e. through counting interaction with friends, family and the wider community) (Cattan 2002). Loneliness, on the other hand, is more difficult to measure and, précised for the purposes of this paper, refers to a subjective state, the ‘unwelcome feeling of lack or loss of companionship’ (Cattan et al. 2005). It is likely that it is this important distinction that has led local authorities to develop indices which focus on identifying social isolation, rather than loneliness.

The above discussion illustrates that whilst social isolation can be linked to a number of different concepts it measures a particular condition and should be treated as a distinct concept in its own right. Furthermore, whilst research evidence suggests that terms such as loneliness and social isolation should not be conflated, it has nevertheless been shown that they are interrelated, as evident with the point raised above that the latter can provide an early indication of the former. Notwithstanding this discussion, this paper is concerned exclusively with social isolation, as this forms the primary focus of the indices discussed.

A final point to make at this juncture is that social isolation as a concept has arguably become subject to a process known as ‘nominalisation’, whereby the action element of ‘being isolated’ (a verb), becomes a noun, a label to describe a particular group. It is suggested by Peace (2001: 21) that when nominalisation occurred with the term social exclusion, individual agency was, at times, lost as the person experiencing it became invisible, or ‘categorised’. It is argued that whilst developing a set of risk factors can be a useful way of identifying potential communities which may benefit from intervention, it is important to ensure the individuals who make up an index based on predetermined categories retain their identity.

### How is Social Isolation Measured?

Before we look at the social isolation indices being developed by some local authorities, it is important to provide an overview of existing research on how social isolation can be measured. There is no consensus as to the most accurate way of measuring social isolation, or indeed the opposite condition ‘social connectiveness’ (Zavaleta et al. 2014), which is unsurprising due to the broad range of psychosocial elements that make up individuals. Thus no index can hope to feasibly capture all factors that may be present in a person who experiences social isolation. However, particular ‘triggers’ are frequently observed through research, which mainly relate to personal characteristics such as age, income or ill health. In many cases these factors tend to overlap; for example whilst the oldest old and ill health are often listed as risk factors of social isolation, the oldest old are statistically more likely to also experience ill health (Local Government Association 2016).

A further issue is that individual indicators of social isolation, such as those relating to socio-demographic, socio-economic and personal circumstances (i.e. health, living alone), are inextricably linked to wider, external ‘community’ or ‘environmental’ factors. This may range from availability of facilities or opportunities in the local neighbourhood, to political priorities in areas such as welfare and health policy. It is therefore important to view the factors discussed in this section as potentially interacting with each other, rather than treating them as unrelated. Triggers will also be shaped by a person’s individual life course trajectory (Durcan and Bell 2015), particularly by significant life events, such as bereavement or retirement (however, not all studies link retirement to social isolation). The following subsections provide an overview of the indicators, which are identified in existing research as contributing to social isolation, broken down into ‘individual’ and ‘community’ level factors.

#### Individual Factors

Several risk factors of social isolation are identified in existing literature based on ‘individual factors’ or ‘personal characteristics’. For example research suggests that older people who live alone (due to widowhood, separation, no partner, or a partner being admitted into a care home) are more likely to be socially isolated (Bartlett et al. 2013; Cornwell and Waite 2009a, b; Durcan and Bell 2015; Jivraj et al. 2012; Victor et al. 2012). Findings also show that being a carer can put someone at increased risk of social isolation (Brennan et al. 1995; Findlay and Cartwright 2002; Durcan and Bell 2015), with the Carers UK (2015) ‘State of Caring’ survey finding that eight out of ten carers feel isolated or lonely due to caring responsibilities. There are also links between low income and higher levels of isolation (Bolton 2012), with ELSA (English Longitudinal Study of Ageing) data showing a far higher likelihood of a person in lower wealth quartiles being socially isolated (Jivraj et al. 2012). Whilst the direction of causation is not always clear, numerous studies also support a link between ill health and social isolation (Jivraj et al. 2012; Durcan and Bell 2015), identifying those with depression (Durcan and Bell 2015), physical ill health (Findlay 2003; Gardner et al. 1999), and those with dementia (Kane and Cook 2013) as more likely to be assessed as social isolated. It has also been found that the ‘oldest old’ tend to experience higher levels of social isolation than the ‘youngest old’ (Cornwell and Waite 2009a, b; Jivraj et al. 2012).

Looking at how social isolation and gender interact presents a more complex picture (Jivraj et al. 2012). Social isolation among men is becoming a growing issue due to an increase in life expectancy, and resulting higher numbers of older men living alone due to widowhood, especially as research shows that older men who live alone are particularly at risk of social isolation (Flood 2005; Bartlett et al. 2013). Research also suggests that men may be less likely to get involved in group based activities, which are often used to tackle social isolation (Beach and Bamford 2015; Milligan et al. 2015).

#### Community Level Factors

Alongside these individual factors, ‘community’ or ‘environmental’ factors are also linked to the prevalence or likelihood of social isolation. For example community assets such as health services, local centres, shops, along with architecture and urban planning (Durcan and Bell 2015) can all affect levels of social isolation. The same can be said of the existence of adequate and safe public spaces, such as pavements and lighting (Durcan and Bell 2015; Marmot et al. 2010). While conversely living in deprived urban areas, or areas where crime is a concern may negatively impact on the development or maintenance of social relationships (Scharf 2002). Other studies have found that lack of access to public or private transport can increase the risk of social isolation (see Russell and Schofield 1999), with ELSA data linking (lack of) access to transport to detachment from civic, cultural and leisure activities (Jivraj et al. 2012).

With regard to housing tenure, a study carried out in the 1970s found people who lived in blocks of flats reported higher levels of social isolation than those who lived in houses (Moore 1975), and a more recent study linked high rise buildings to lower levels of satisfaction (Gifford 2007). High rise accommodation is often connected to a number of negative issues such as: fear; dissatisfaction; stress; behaviour problems; suicide; and reduced helpfulness of neighbours. At a societal level it is argued that high rise living can lead to an increased burden on existing services and infrastructure, worsening traffic problems, and damaging the character of neighbourhoods due to exacerbating problems such as crime, and an absence of ‘community spirit’ (see Gifford 2007; Broyer 2002). One review also concluded that residents in high-rise blocks have poor social relationships (Korte and Huismans 1983). However other research has found that friendships within high rise blocks can be developed (see Gifford 2007). Overall, housing type has been found to have an impact on social networks and community cohesion. However, as with other potential indicators, assessing the impact that high rise accommodation may have on social isolation is difficult, as non-architectural factors also come into play. For example people who live in this type of dwelling may also differ in terms of their socio-economic status (Gifford 2007), or may be more socially isolated due to differing availability of resident activities.

## Social Isolation Indices Developed by Local Authorities

Due to the aforementioned policy focus around reducing social isolation, and in light of the desire to adopt a preventative approach, some local authorities are developing indices in an attempt to map areas where residents are predicted to be most at risk. In broad terms, each index includes a range of social indicators (numerical measures) to identify the prevalence of social isolation. There are no nationally validated measures of social isolation and consequently individual local authorities have developed various differing local level indices. A number of existing indices have been identified online by the authors. This paper considers social isolation indices developed by local authorities in: Barnet, Bristol, Essex, Gloucestershire, Leeds, Luton, Maidstone, Medway, Somerset, Surrey, and Wirral (a few were identified which claimed to measure both social isolation and loneliness, these were not included so as to avoid confusion). All the above-mentioned local authorities have identified indicators through geo-demographic segmentation tools which provide lifestyle data at postcode level. Experian Mosaic is often used for this purpose (a customer segmentation tool, which is used to map community residents through a range of criteria, including social isolation, to help organisations, including local authorities, to locate their target audience). A few indicators, in addition, have been gained through information held by the authorities themselves (such as social care referrals). In most cases the indices are at relatively early stages of development (i.e. mapping has occurred, but further work around how to use this information is still taking place). Most indices are concerned with all age groups, some include a large range of indicators (up to 18), whereas others have used only a few (the lowest being three). Table one provides an aggregated overview of the main indicators included in the indices examined.

Most of these local authorities have produced maps at neighbourhood/ward level; two have developed indices at two levels, including at neighbourhood/ward level, and household level. For the maps produced see Bristol City Council (2014), Kinsella (2015), Leeds City Council (2014), Luton Borough Council (2014), Maidstone Borough Council (2014), Medway Council (2013), Naylor (undated) (**XXXX**), Parsons (2016), Somerset Intelligence (2016) and Surrey County Council (2015). The individual indicators (many of which have been assigned weighted scores) have been aggregated, with the highest scoring households or areas being used to map potential ‘hotspots’ of social isolation (i.e. where social isolation is likely to be highest or more prevalent). In some cases local authorities have broken down their indices further. For example one local authority has assessed how overall social isolation scores are affected when housing tenure is taken into account, comparing the relative score of social housing tenants to those who reside in other tenures (though none of the local authorities included housing tenure (or type) in their main indices). Most local authorities have mapped social isolation for all ages, two of which produced maps for those under and over 65 years of age.

As a wide range of factors have been found, in existing research, to be indicative of social isolation, the local authorities have inevitably been required to apply some form of inclusion criteria, leading to some variables being excluded. For example one local authority acknowledged limitations in their index due to excluding indicators relating to income and ethnicity. However, in most cases, limited justification for which indicators were used or excluded are included in reports, though it is assumed that availability of data would have had at least some influence on this. In all cases the indices refer mainly to the individual level indicators mentioned above, such as income, education, living alone, health, widowhood, retirement and ill health, with ill health being adopted as one of the most prevalent indicators across the local authorities. Other factors, such as belonging to an ethnic minority group, not having English as a first language, or access to transport, was only included in a small number of local authority indices (see Table 1). The indices also pay less attention to ‘community’ level factors such as housing type (e.g. living in high rise flats), level of crime, local services, and other environmental variables. However, these wider factors are less amenable to being placed in an index (though one authority included anti-social behaviour prevalence and fear of crime). In some cases community level data may not be available, and the relationship between some of these variables and social isolation is not always clear (i.e. high rise housing may increase or decrease social isolation, according to a number of other factors).

**Table 1:** Indicators included in the social isolation indices of 11 local authorities

Personal circumstances	Health	Attitude/social networks
Age (including 65 + or 75 +) Divorce/separation Single pensioner Widowed Retired Live alone Non-white Gender (female) People in the household not having English as a main language Deprivation/low income Struggling financially Household annual income below £20,000 Pension credits Unemployment Less educated (no further education, no degree) A household without private transport Internet -never used Received an adult social care referral Having an unpaid carer within the household Being a lone parent with dependent/non-dependent children % of people aged over 65 in area	Mental ill-health (depression, self-harm, anxiety) Poor mobility Chronic health condition/permanently sick Long term health condition Dementia/cognitive decline Being overweight Vision loss/impairment Hearing loss/impairment Urinary incontinence Alcoholism in men Poor general health Poor self-care	Unlikely to meet friends/family regularly Unlikely to interact with neighbours Have no-one to help in a crisis Not satisfied with social life Having an attitude which thinks little can be done to change life Having someone who will listen Have someone you can relax with Access to the internet Access to Facebook Perception of crime in area Reports of anti-social behaviour

Very similar indicators have been amalgamated, meaning precise wording may be different on some indices

The 11 Local authorities include: Barnet (Naylor undated **XXXX**); Bristol (Bristol City Council 2014); Essex (Dawkes and Simpkin 2013); Gloucestershire (Parsons 2016); Leeds (Leeds City Council 2014); Luton (Luton Borough Council 2014); Maidstone (Maidstone Borough Council 2014); Medway (Medway Council 2013); Somerset (Somerset Intelligence 2016); Surrey (Surrey County Council 2015); and Wirral (Kinsella 2015)

Most of the local authorities which have developed a social isolation index state that they aim to utilise it to develop solutions at a community level, and to better target services that prevent, or alleviate isolation. However it should be noted that progress is still at early stages. Furthermore, qualitative work to support the indices has been limited. For example, one local authority carried out focus groups with a range of individuals and stakeholders to test their index. Another local authority reported that they are looking to carry out focus groups in the future, but no details as to when this may happen could be found. A third local authority recognised that local experience will be a ‘useful yardstick’ for which to further consider the findings, but, as far as the authors are aware, as yet there is no published evidence of this taking place.

With the exception of Leeds City Council, none of the local authorities have attempted to validate their social isolation indices by carrying out qualitative research with individuals living in specific neighbourhoods.

### Leeds City Council Social Isolation Index: An Overview

Leeds City Council commissioned the authors to carry out a qualitative study of social isolation in the city to help assess the usefulness of social isolation indices. Two social isolation indices, aimed at people aged over 65, were developed by Leeds City Council Public Health Intelligence. The first one assessed scores at ward level, and the second at street level (i.e. to the individual address). Both indices include data for a number of ‘sub-groups’ of older people and, aside from Adult Social Care referrals, all are based on the Acorn consumer classification index. The indicators include:

- non-white
- have at least one long term health condition
- diagnosed as having dementia
- widowed
- in receipt of pension credits
- received an Adult Social Care referral (ward level index only).

The remainder of this paper outlines the findings of novel empirical research carried out by the authors to assess the validity of the second Leeds City Council social isolation index (street level), providing original research findings, something which has not yet been undertaken elsewhere.

## Methods

A qualitative ‘sense check’ of the validity of the second index developed by Leeds City Council (which was developed at household level and therefore did not include individuals who had an Adult Social Care referral) was carried out by the research team. Given that this index could potentially pinpoint specific addresses which are more at risk of social isolation than others, the council were keen to assess the validity of this index, rather than explore the usefulness of the first index. This household level index produced a list of addresses predicted to contain a person over 65 at risk of social isolation. In conjunction with Leeds City Council the research team selected three of the 33 wards in the city to carry out the sense check. Two of these wards were predicted by the index to have high levels of social isolation, whereas the third was assessed as having a low overall level of social isolation.

The sense check involved carrying out interviews with people aged over 65 in the three wards, including those who were living at addresses identified by the index at risk of social isolation and those who were not. The interview schedule incorporated nine questions designed to assess the level of social isolation experienced. For the purposes of exploring the Leeds Social Isolation Index the questions include some objective measures of social, leisure, and wider networks, with other questions focused on perceptions of isolation, such as whether the older person perceives themselves to be isolated or lacking companionship (so touching more upon what is generally described as ‘loneliness’), more specifically the interview schedules asked questions on:

- Social contacts which looked at face to face and other forms of contact with friends and family (three questions).
- Participation in community level networks, for example contact with neighbours and participation in local events (two questions).
- Participation in leisure activities, which may include activities in the local community, but also captures information around wider activities (one question).
- Perceptions of isolation and companionship (three questions).

The aim of the interviews was to enable us to assess qualitatively the robustness of the index. We used the interviews to firstly ‘count’ incidences of social interaction and participation (through the use of close-ended set questions), allowing us to assess how useful the index was. This was then supported through in-depth discussions with older people about their experiences of social isolation (through the use of open questions). Where appropriate responses are recorded as a percentage for the set questions, but for the open ended questions, which generated more qualitative discussion, recurring themes and illustrative examples are drawn upon. The research team did not audio record the interviews, and for this reason illustrative quotes are not provided, as it did not seem appropriate to do so without having the ability to listen back to verbatim accounts. Whilst the researchers were out in the field detailed observational notes were taken, including information about the nature of the areas visited, and details provided by local residents who were not eligible or were unwilling to be interviewed. The aim of this was to inform future research of this kind, and identify any particular relevant issues.

A qualitative sense check was selected for this study as opposed to a larger scale quantitative analysis. A quantitative analysis could have been developed by conducting a large scale questionnaire survey of the households both included and not included in the social isolation index. This would have enabled a statistically rigorous testing of the index. However, for a number of reasons, it was decided that a qualitative sense check was a more appropriate methodological approach on this occasion. It was felt that a qualitative sense check would enable the older people themselves to identify issues that they feel influence their experience of social isolation which we would not necessarily have uncovered through a quantitative survey. It was also recognised that the index developed by Leeds City Council was a first attempt to map and identify those most at risk from social isolation in the city but that further work was likely to be needed to refine the index. Given the time and resources required to carry out a large scale quantitative analysis, it was therefore more appropriate to carry out a smaller in-depth piece of qualitative research which could be completed fairly quickly and with a relatively small budget. It was envisaged that by carrying out a qualitative sense check, findings could be disseminated back to Leeds City Council in a timely manner and they could then amend the index as appropriate (which is precisely what the Council did as a result of the research findings). A next future step for this research would ideally be to subject the Council’s refined index to further testing through a large scale quantitative questionnaire survey of households in the city, supplemented with a qualitative study with a selection of households, to help explore the findings.

The research team delivered letters to 682 addresses (of which 296 people were included on the ‘at risk’ list) inviting the older residents to take part in an interview. At least one week later researchers called at the doors of households who had received a letter, asking if they would be willing to participate in the research. A card was left for residents who did not answer the door when researchers initially called (providing the property could be accessed). The card included a brief reminder of the purpose of the research and gave residents the option of getting in touch with the research team by telephone to arrange an interview at a convenient time. The research team visited all properties at least once in order to request an interview. Many of the households situated in blocks of flats were visited two or three times, on different days and at different times as no-one was home on the initial visit(s).

In addition to the interviews with residents, focus groups and one-to-one interviews were carried out with various stakeholders in each of the three localities comprising those who either have an interest in, or work with, older people in each area. This included: representatives of local voluntary organisations; community workers; sheltered housing wardens; councillors; GPs/nurses; pharmacy staff; social workers; housing officers; and occupational therapists. The purpose of engaging with stakeholders was to gain a contextual understanding of some of the issues that they encounter, and to discuss if they are aware of particular areas/streets in which isolated older people tend to reside. Whilst the stakeholder discussions elicited important information, this paper primarily focuses on the views of older people themselves, though stakeholder views are touched upon when ward level findings are discussed.

### Accessing Participants

Of the 682 letters delivered, a total of 90 (13%) older people across the three wards agreed to be interviewed, of whom 41 were men (46%) and 49 were women (54%). The households of the residents who were interviewed were checked against data from the index provided by Leeds City Council, and 59 (66%) of those who were interviewed were included on the ‘at risk’ list and 31 (34%) were not. The time that each of the interviews took ranged from 15 to 45 min. Detailed notes were taken during each interview which were inputted into an Excel spreadsheet, and levels of social isolation were assessed based on responses to individual questions (for example a ‘count’ of number of contacts and activities, of various types, were inputted).

We were keen to ensure that a range of older people were interviewed, and their views sought. Yet the research team encountered a number of difficulties gaining access to older people in the three wards visited. Nearly half (305/682) did not answer the door; in some cases this was despite returning on a number of occasions, at different times of the day. 42% (287),^1^ declined to be interviewed, two-fifths of whom (112) were identified as ‘at risk’ of social isolation according to the index. Where residents answered the door but declined to be interviewed a number of different reasons were provided, which are listed in Table 2.

**Table 2:** Explanations for residents declining to participate in the research

Reason for declining to participate in the research	Number of cases		
	Not on the index	On the index	Total (%)
Residents not meeting the age criteria (i.e. being under 65)^a^	51	14	65 (36%)
Not interested	20	12	32 (18%)
Research not relevant to them	1	5	6 (3%)
Warden advised not to interview	40	0	40 (22%)
Too busy	10	12	22 (12%)
Ill-health/disability/bereavement	6	6	12 (6%)
Fear for safety/security	1	1	2 (1%)
Other	1	0	1 (1%)

^a^This was due to information gathered being incorrect—which may have been the case for a number of reasons, such as the details provided being out of date

Differences in response rates across the three wards were observed, which may be linked to factors specific to the localities visited. For example residents who lived in a ward reported as having high levels of crime were the least likely to answer the door. However, it must be stressed that as the research team did not get to speak to these particular residents, these issues could not be explored in further detail. In some cases when residents did not answer the door, someone appeared to be inside the property; either noises were heard inside (such as footsteps or a television), or mobility scooters were parked outside. This occurred mostly in a particular high rise block predicted to have a high proportion of socially isolated older people. A potential link between willingness to respond and fear of crime is supported by Scharf and Jong Gierveld (2008), where examples were provided of people who had been a victim of crime being unwilling to respond to callers unless a visit had been pre-arranged.

There is a concern that the research team may have missed frailer isolated older people, or that they may have been less likely to participate in the research. In some cases, for example, family members got in touch with the research team, or spoke to researchers when they were out interviewing in the communities, to say that their relatives were too ill or frail to take part (most commonly due to recently being discharged from hospital, or having a cognitive impairment, such as dementia). The son of one resident, living in a block of flats visited, advised that his mother was over 65 and isolated due to ill-health, but he felt unable to allow the researchers to interview her due to her health issues. In another example, a member of the research team had secured an interview with a resident who said that she felt she had limited support. However, later that day a relative got in touch with the research team to cancel the interview, stating that the older person in question often became confused, so lacked capacity to take part. Both these cases represent examples where the older person may well be isolated, but where we were unable to explore further. On the other hand there was also a concern that older people who were particularly busy or active were not always reached in the research either, because they were not at home when the interviewers called, or because they felt that the topic was of no relevance to them. This means that participation in community networks or leisure activities, in particular, could be over or underrepresented, although each may cancel the other out.

## Using an Index to Identify Social Isolation in Three Localities

This section considers the key findings gained from the interviews across the three localities. It is important to note that the number of interviewees, at 90 across the three wards, does not necessarily allow for robust comparison between those who are identified ‘at risk’, and those *not* identified ‘at risk’. However, the aim was exploratory, to gain a deeper understanding of the kinds of contextual information that interacting at the ‘street level’ can provide. The interview findings are in fact being utilised by Leeds City Council to inform further development of its index.

### Participation in Outside Activities/Community Networks

In total just under one-fifth of those interviewed could be considered isolated from community networks. This means that there was no-one other than family or friends they could rely on, and they did not participate in any clubs, activities or groups in the area. There was a correlation between participation and disability (discussed further below), with over a quarter giving this as a reason (or stating that they were caring for a disabled person) for not getting involved in activities in their area. When looking at findings by locality, differences in levels of isolation from community networks were observed, with responses ranging from nine per cent to 20 per cent. The ward with the highest reports of crime and anti-social behaviour had the highest levels of community involvement, suggesting crime did not necessarily have an adverse impact on community engagement. However, this area had a fairly wide range of local activities or activities within high rise blocks, which ran during the daytime. Concerns among residents related to fear of visiting local shops, or going out at certain times of the day. A further issue was that residents in this area were the least likely to answer the door, so the findings may underrepresent those who are less willing to engage in community networks due to a fear of crime.

The extent to which people engaged in community activities was, in part, determined by the availability of activities in their immediate area and their ability to access them. For example availability of activities within blocks of flats could be particularly important for those with a disability, who may struggle to travel even short distances. Indeed, some older people advised that they were unable to take part in activities outside their homes due to disability. Of the 23 blocks of flats visited across the three localities (12 of which were high rise, and 11 low rise) all except two either ran community led activities and events, or older people reported being able to access something closeby. Most of the older people interviewed in a block of flats, where no activities took place, had higher levels of isolation from community networks due to the lack of facilities (e.g. only two out of the 11 interviewed were involved in any community network). There were also verbal reports that, due to previously run community events being disbanded, residents were more socially isolated as they now had less contact with their neighbours. A few older people felt that the localities they lived in did not have the ‘community spirit’ they once did, and that they no longer knew all their neighbours. However, in some cases residents did not get involved in community activities as what tended to be on offer (i.e. bingo, coffee mornings), did not interest them. This point highlights the heterogeneity of older people, and also the importance of talking to a broad range of residents to ensure that solutions are based around interventions or activities that they will actually want to take part in.

Respondents were asked about their involvement in leisure pursuits outside of their immediate communities, including visits to the cinema, art gallery, museum, theatre, sports events, concerts, going out for drinks or a meal at least once in the last few months. Of those who had not taken part in leisure pursuits in the last few months, most stated that this was mainly due to disability, and one due to caring responsibilities. Transport also appeared to be a factor, with some residents in one ward suggesting a more regular bus service into town would help them participate in more leisure activities. The ward where the highest numbers of residents were involved in leisure activities all reported very good transport links.

### Self-Reported Isolation

Only six of the 90 interviewed (7%) referred to themselves as being isolated ‘all’ or ‘most’ of the time; the same number reported lacking companionship. As expected, the interviewees who reported lacking companionship lived alone (though all saw friends or family in a typical week). In all except one case perceived isolation did not appear to be related to the number of social contacts or activities interviewees were involved in. Alongside this a few interviewees who reported that they felt isolated or lacked companionship had, on the face of it, a wide social and community network. Conversely, just under a third of interviewees reported preferring to be alone, choosing not to get involved in outside activities. Related to this, some older people reported that whilst there were a number of activities in their block of flats and/or in the surrounding area, a number of residents in the block did not participate. Although in one case it was suggested that this was because it was harder to reach more isolated people to make them aware of events, others believed this was a preference, as some people would rather be alone. This was supported by some interviewees, who stated that they did not want to become involved in a close knit community, suggesting other residents were ‘cliquey’ or ‘nosey’. These findings demonstrate that someone who scores highly for social isolation will not necessarily feel lonely. It also highlights the need to take into account, when developing interventions to reduce social isolation, that people who score highly may nevertheless be reticent to get involved.

### The Usefulness of the Indicators Included in the Social Isolation Index

Based on the older people interviewed across the three localities, only disability appeared to impact on levels of social isolation relatively independently of community led factors (however, as only one person interviewed belonged to an ethnic minority group, the research team is unable to comment on this indicator). For example one interviewee reported that as a result of motor neurone disease, her health fluctuated; when she felt well, she went out and socialised frequently, but when unwell she felt more isolated due to being unable to take part in the activities she normally enjoyed. Conversely, a few older people reported that a successful medical operation (i.e. hip replacement) or mobility scooter had made a positive difference to their ability to access community and leisure activities. Yet whilst on face value, disability can be viewed as an individual level indicator, social interaction for people who reported health conditions was strongly linked to broader factors, such as housing tenure or community led facilities. One respondent, a wheelchair user, relied on a neighbour to help her get outside due to living in a high rise flat, and if the neighbour was not available, she was unable to get outside. Another interviewee, who lived on the top floor of a high rise block, felt that even though she was able to get out and socialise as she was fit and healthy, if this was not the case, the accommodation would be isolating as she does not see her neighbours. Yet another reported being unable to participate in the activities that she used to enjoy due to disability, and relied on community activities which were closeby. Another factor which could be explored further, based on the interviews, is gender; the findings showed that overall men were less likely than women to get involved in community led activities (with 61% of women involved compared to 50% of men), though they were slightly more likely to take part in leisure activities (62% of men compared to 57% women) (these findings are consistent with those identified by ELSA, discussed earlier).

As touched upon above, place based factors, such as property type or locality, rather than individual factors or personal characteristics, were often referred to in the interviews as significant. For example, it was found that two blocks of flats in the same street reported being isolated from community networks due to the cessation of resident activities, whilst other blocks on the same street still ran activities, thus reported higher levels of community involvement. However, despite these different experiences all residents across the blocks shared a similar score on the index, even though community participation meant actual experiences of isolation were quite different. This is due, as highlighted earlier, to the focus on including individual level indicators/personal characteristics within the index. A few interviewees reported that they had difficulties attending certain activities or events due to either infrequent or unsuitable transport options in their area. Yet others attributed a lack of participation to low levels of neighbourliness in their respective areas (which is returned to below when we discuss community networks). Some reported feeling lonelier in the evening, yet nevertheless preferred to stay at home due to disability, becoming tired, or having concerns around safety.

It was further found that in a ward where some interviewees (and nearly all local stakeholders) reported high levels of crime and antisocial behaviour, indicators such as income appeared secondary to fears around safety. Some examples included reports of young people drinking outside blocks of flats, and older people being afraid of going out after dark. Properties in this area were also the most likely to have a locked steel gate in front of the main entrance door. One interviewee informed the interviewer that she had left her front door unlocked whilst she unpacked her shopping, and that someone had come into the hallway during this time and had stolen her handbag. Some interviewees, based at two high rise blocks in the area, suggested they felt less safe since younger people have been allowed to move into the block of flats, and also complained of anti-social behaviour, such as drinking in communal doorways and urinating in the lifts. Another complaint was that there was little police presence, which may be linked to what focus group participants referred to as under-reporting of particular types of anti-social behaviour. As highlighted above, residents in this ward were the least likely to answer the door to researchers; whilst it cannot be asserted with certainty, there may a link between this and reported fear for safety in the area.

## The Validity of an Index to Measure Social Isolation

Our research has demonstrated the importance and additionality of carrying out a qualitative sense check to supplement indices designed to assess those at risk from social isolation. A particularly valuable element of interviewing people in neighbourhoods where social isolation was estimated to be high was having the opportunity to ask both residents and stakeholders their opinions and experiences. This provided a real sense as to how experiences can differ both within and between localities and micro neighbourhoods, with experiences not just influenced by personal circumstances such as having a health condition or being in receipt of pension credits but by a wider range of environmental and cultural issues. The findings lend support to the view that social isolation must be viewed as a multidimensional concept, highlighting the interplay between individual characteristics, circumstances, types of social isolation (i.e. on the basis of social, leisure or community networks), and the extent to which these are influenced by local environments (i.e. housing type, local activities, fear of crime, transport). This should be considered alongside literature which has demonstrated the relationship between social connectiveness and wider economic, political and cultural contexts. This therefore has important connotations for the usefulness of an index to identify and measure social isolation. Without an understanding of the ‘multifacetness’ of social isolation, and how factors may be shaped by factors unique to localities, an index alone is unlikely to be sufficient.

A qualitative analysis can also assist in identifying targeted solutions shaped around needs unique to local areas, something which an index alone cannot achieve. For example it was found that environmental factors, both at ward and micro locality level (i.e. blocks of flats situated on the same street), were generally more indicative of social isolation than income, and even disability, in some cases. The research further helped to contextualise the varied needs of residents, discouraging the adoption of a ‘one size fits all’ approach. Ultimately, it makes sense to consider place based factors when research tells us that adopting a community level approach provides the most effective route to preventing social isolation (Hole 2011; Marmot et al. 2010); indeed local authorities themselves acknowledge this in their strategies. The results of the sense check also drew attention to the fact that tackling social isolation requires a ‘whole area’ approach, which does not focus exclusively on tailored activity to reduce social isolation. A good example of this was provided in the ward where crime and anti-social behaviour was viewed as a problem, where strategies aimed at getting younger people involved in the community were seen as an effective way of ensuring older people felt more comfortable integrating into the local community.

Carrying out supplementary qualitative interviews may also help temper the somewhat ‘top down’ nature of using an index alone, which runs the risk of disempowering those it seeks to identify. That is, without adding the voice of those being measured to indices which rely on predetermined categories, the identification of social isolation in this context becomes reminiscent of the ‘nominalisation’ process discussed in the opening sections of this paper. As uncovered during the interviews, people who were identified ‘at risk’ by the index did not necessarily fit neatly into the category of ‘socially isolated’; indeed those not included ‘at risk’ could be just as isolated as those who were identified ‘at risk’. Conversely, many older people who were identified ‘at risk’ on the index expressed high levels of social connectiveness. Related to this, the sense check assisted in assessing the robustness of the indicators applied by Leeds City Council, which led to recommendations around potential ways of refining the existing index, which are currently being considered.

Whilst conducting supplementary qualitative fieldwork at a neighbourhood level is recommended, it is not without challenges, with issues around engaging local residents being the most pertinent. For example, attempting to gain the views of some groups, such as the very isolated, those with particular health problems, or those in high rise blocks, proved difficult. Therefore local authorities should consider access issues carefully and also think about how to ‘sell’ the benefits of participation, such as to those who are unwell, or to those who do not class themselves as socially isolated. It is recommended that ‘trusted’ organisations within local communities, such as GPs, health visitors, or local community organisations are approached, as they may be able to access particular hard to reach groups through developing a relationship over time. This is especially important in light of suggested links between fear of crime, and willingness to respond to unknown callers. It may also be worth getting in touch with local carers’ organisations and similar, in an attempt to get access relatives or carers of those with sensory or cognitive impairments, who can be present and support the person during interview.

A further consideration is how to access particular groups within communities, such as ethnic minority groups, which research evidence has shown can be more vulnerable to social isolation; yet barriers, such as those related to language, may make it more difficult to capture their views (Findlay and Cartwright 2002). It is reiterated that due to the low number of interviewees overall, some of the more specific findings discussed in this paper should be treated with caution. However, the research does effectively demonstrate the ways in which qualitative interviews add richness to test and verify the validity of an index. On a final note it is recommended that further work is carried out to explore the potential for developing a national level social isolation index, as at present there is considerable overlap between the indices being developed by local authorities. A national index can then be used as a broad guide, with further contextual work being undertaken at the local level. An undertaking such as this may require central direction, both in terms of steering and funding the work required to carry this out.

## Conclusion

This paper has provided a novel contribution to the emerging approach of developing indices to identify those most at risk from social isolation. It has reported on a unique qualitative study used to sense check a social isolation index developed by a northern local authority and argues that this kind of qualitative analysis ensures that indices are able to capture the multifaceted nature of social isolation, which often extend beyond individual and household indicators to include issues unique to local spaces. The need to incorporate the views and experiences of those subject to the indices is further reinforced as, without this, people remain ‘invisible’ and local authorities cannot be confident that they have accurately identified the location, incidence or triggers of social isolation in their respective areas.

It is therefore recommended that a qualitative sense check be incorporated into existing and newly developing indices. Given that a number of local authorities are developing such indices there may well be some merit in exploring the potential for developing a national level social isolation index. Perhaps respective areas can then be supported to create a ‘toolkit’ which local authorities and various third sector organisations can use to supplement the index and apply to their own unique local contexts. This could not only aid identification of people and areas most at risk of social isolation, but it may provide a more effective way of applying solutions in areas where social isolation is assessed as being high. For example the solutions will be different if a social isolation ‘hot spot’ is in a rural area with limited transport, or an urban area where anti-social behaviour is rife.

## Footnotes

1 This number includes 40 households which were not revisited on the request of the warden of sheltered accommodation.
